# Recovery and resilience of tropical forests after disturbance

**DOI:** 10.1038/ncomms4906

**Published:** 2014-05-20

**Authors:** Lydia E. S. Cole, Shonil A. Bhagwat, Katherine J. Willis

**Affiliations:** 1 Department of Zoology, Oxford Long-term Ecology Laboratory, University of Oxford, South Parks Road, Oxford OX1 3PS, UK; 2 Department of Zoology, Biodiversity Institute, Oxford Martin School, University of Oxford, South Parks Road, Oxford OX1 3PS, UK; 3 Department of Geography, Faculty of Social Sciences, The Open University, Walton Hall, Milton Keynes MK7 6AA, UK; 4 Department of Biology, University of Bergen, P.O. Box 7803, N-5020 Bergen, Norway; 5 Royal Botanical Gardens, Kew, Richmond, Surrey TW9 3AB, UK

## Abstract

The time taken for forested tropical ecosystems to re-establish post-disturbance is of widespread interest. Yet to date there has been no comparative study across tropical biomes to determine rates of forest re-growth, and how they vary through space and time. Here we present results from a meta-analysis of palaeoecological records that use fossil pollen as a proxy for vegetation change over the past 20,000 years. A total of 283 forest disturbance and recovery events, reported in 71 studies, are identified across four tropical regions. Results indicate that forests in Central America and Africa generally recover faster from past disturbances than those in South America and Asia, as do forests exposed to natural large infrequent disturbances compared with post-climatic and human impacts. Results also demonstrate that increasing frequency of disturbance events at a site through time elevates recovery rates, indicating a degree of resilience in forests exposed to recurrent past disturbance.

## Abstract (web summary)

Forest recovery after past disturbances can help to understand how forests will respond to future scenarios. Here, the authors analyse palaeoecological records from tropical forests and find that Central American and African forests have recovered faster after disturbances than those in South America and Asia.

## Graphical abstract

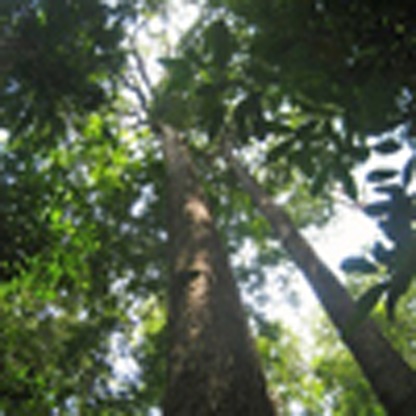

Tropical forests are recognized globally for the important ecosystem services that they provide. Knowledge of recovery rates and responses of tropical forest to past forms of disturbance may facilitate our understanding of the capacity of these ecosystems to respond to present and future events. Although it is generally accepted that if left long enough, tropical rainforest will recover^1^, there is much debate about the nature of forest re-growth and the time taken for it. Re-establishment of forest to approximate pre-disturbance levels, as reported predominantly from single-site^2^,^3^ and chronosequence studies^4^, typically requires 20–200 years, depending on the variable measured^5^,^6^,^7^. In *tierra firme* forests of the Amazon basin, for example, levels of species richness comparable to those found in mature forest are re-established in a quarter of the time taken for equivalent forest biomass^4^. However, these studies make no explicit reference to the history of disturbance in the described location, which could account for significant variation in rates of recovery between sites, even among those from similar abiotic and biotic settings. Through examining recovery rates from 283 past episodes of disturbance reported in palaeoecological records, we identified the factors that affected responses of tropical forest ecosystems to perturbation through time. We addressed two fundamental questions: first, how quickly has forest re-established after deforestation events in the past?, and, second, what factors—disturbance type, geographical location, frequency of disturbance events—affect recovery rates?

To determine trends in reforestation rates of tropical forests, we conducted a meta-analysis of 71 published fossil pollen records, which focused on four tropical regions: Asia, Africa, and Central and South America. After identifying studies through a literature search using Scopus ( www.scopus.com), exploring both key words, such as ‘tropical forest’ and ‘fossil pollen’, and key authors performing research in these regions, various selection criteria were applied. Data sets were included based on their location (within 23°N and 23°S), evidence of disturbance events (and associated recovery) over the past 20,000 years and good chronological control (^14^C dating); a lack of any of these criteria resulted in the exclusion of the study. From the selected 71 records, each resulting from a sediment core extracted from a specific geographical location, a data set of 283 disturbance events was compiled and analysed, with a quarter of records demonstrating five or more of these episodes. Disturbance events are defined as perturbations that result in a sharp loss of forest pollen in the palynological record and subsequent gain, and include both catastrophic disturbances, where relative forest pollen may decline by > 90% (FD, see Table 1), to smaller perturbations where declines of <10% may occur during a period of relative forest stability or longer-term recovery. Forest recovery is described as the maximum increase in the percentage of forest pollen in the displayed pollen sum after a decline, before a stabilizing point or further decline (F_max_, see Table 1). The focus of this meta-analysis is on the recovery rate (RR) of forest, as defined uniquely in each individual study (see Supplementary Data 1), representing the speed and extent to which this ecological unit re-grows relative to its pre-disturbance abundance (not always equivalent to an undisturbed state), with the assumption that vegetation re-establishment, as inferred through the proxy of fossil pollen, is indicative of sustained ecosystem functionality. The type of disturbance at each event was classified according to information on the published pollen diagrams and/or in the article text (Table 2). The main variables extracted for forest RR calculations are presented in Table 1.

This novel approach to analysing long-term trends in tropical forest re-establishment provides fundamental insights into the functioning and resilience of tropical forest ecosystems. Results demonstrate that forests in the Central American tropics respond more rapidly to disturbance than those in Asia, and that recovery after large infrequent events is more dynamic compared with re-growth post-climatic or anthropogenic impacts. In addition, it is the frequency of past disturbance events that appear to play the most significant role in determining the rate of reforestation in these tropical regions: the greater the rate of disturbance through time, the faster the recovery. These findings suggest that tropical forests, in response to an ecologically-relevant history of exposure to natural disturbances, are adapting to perturbation through time, evolving mechanisms that increase their resilience in the face of environmental change.

## Results

### Rates of recovery

**Figure 1:**
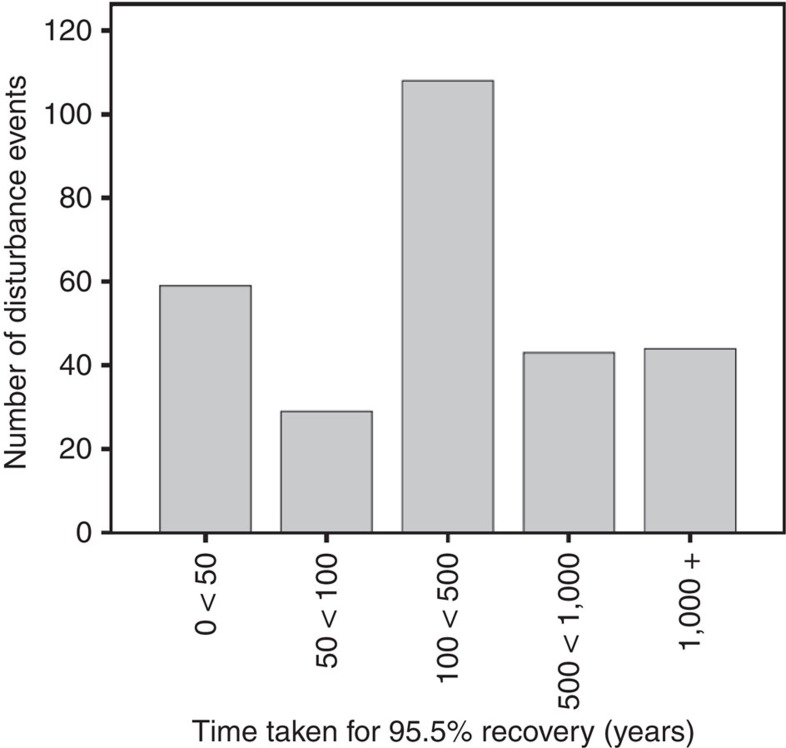
Number of disturbance events versus years to 95.5% forest recovery. The relationship between recorded disturbance events and numbers of years for 95.5% recovery to pre-disturbance forest abundance baseline at each event (*n*=283) is shown. Recovery rates were used to calculate the time taken to a projected 95.5% recovery, that is, the median extent of recovery for the data set.

Results demonstrate that the majority of past disturbances were caused by forest clearing, followed by climatic changes and large infrequent events (*n*=166, *n*=87 and *n*=13, respectively). For the majority of disturbances, forest re-growth stopped before 100% recovery either due to the onset of another disturbance event or the establishment of a lower forest proportion in the landscape (approximated through fossil forest pollen percentages); nevertheless, median forest abundance after disturbance reached 95.5% of pre-disturbance levels (*n*=283). Across disturbance types, recovery rates varied greatly, with projected times to 95.5% forest recovery from the pre-disturbance baseline ranging from <10 to 6,846 years (*n*=283), while the estimated median time was 210 years (*n*=283) (Fig. 1) and average time 503 years (s.d.=768.16, *n*=283).

### Recovery rate versus disturbance category

**Figure 2:**
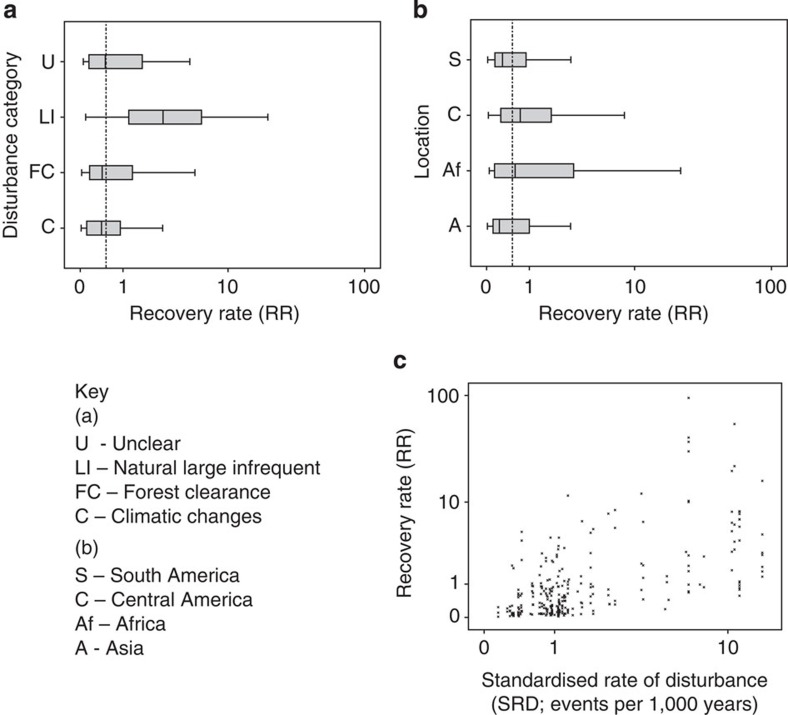
Composite figure showing recovery rates (RRs) of different covariates. Panels display the relationship between RR and (**a**) disturbance categories, (**b**) geographical locations and (**c**) against the standardized rate of disturbance (SRD). (**a**) Box plot of RR for grouped disturbance categories. C, climate-related factors (*n*=87); FC, anthropogenic forest clearance, grouping FC, SC, B, Ag and combinations of these (*n*=166); LI, natural large infrequent events (*n*=13); U, cause of disturbance unclear (*n*=17). (**b**) Box plot of RR for different location groups. S, South America (*n*=85); C, Central America (*n*=111); Af, Africa (*n*=29); A, Asia (*n*=85). (**c**) Scatter plot illustrating the relationship between the RR and SRD. Throughout, RR and SRD are plotted on logarithmic axes to accommodate the variability in these data sets. The vertical line in **a** and **b** represents the median RR for the entire data set, that is, 0.455% relative recovery per year. Shaded areas on **a** and **b** represent the interquartile range and the whisker lines the 95% confidence intervals for each category.

The response rates of tropical forest to natural versus anthropogenic disturbances were not significantly different, though the greatest range in RR across categories was between climatic and large infrequent disturbances (linear mixed-effects model, *P* value=0.072, *n*=283; Table 3) (Fig. 2a). RRs were fastest after large infrequent disturbances (LI) (median=2.84% relative reforestation per year, *n*=13) (see Table 2 for details of RR calculation), and notably slower after climatic changes (C) and human-induced disturbances (FC) (for both, medians=0.41% relative reforestation per year, *n*=87 and *n*=166, respectively).

### Recovery rate versus geographical location

**Figure 3:**
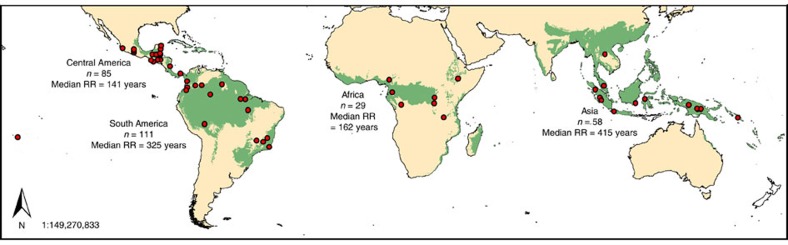
Map highlighting the World’s tropical forest ecosystems and the location of all studies included in this meta-analysis. For each of the four regions, the number of disturbance events (*n*) and median time to 95.5% recovery of pre-disturbance baseline (median RR) are shown. (The WWF Biome of tropical and subtropical moist broadleaf forests is shown in green^40^.)

The broad geographical region in which the forests reside did significantly affect the RR (Figs 2b and 3). In general, forest ecosystems in the Central American tropics recovered significantly faster than those in Asia (linear mixed-effects model, *P*-value=0.027, *n*=283; Table 3), the latter of which recovered the slowest, with median times for 95.5% recovery of 141 years (*n*=111) and 415 years (*n*=58), respectively. In general, RRs were also significantly faster in African forests compared with those in South America and Asia (linear mixed-effects model, *P*-value=0.041 and 0.009, *n*=283, respectively; Table 3). However, latitude did not appear to have a significant effect on RR (linear mixed-effects model, *P*-value=0.111, *n*=283; Table 3), along with the longitudinal and altitudinal setting of the forest (both excluded from Model I (Eq. 1) when *P*-values>0.100 were demonstrated in initial regression analyses, *n*=283).

### Frequency of past disturbance

**Figure 4:**
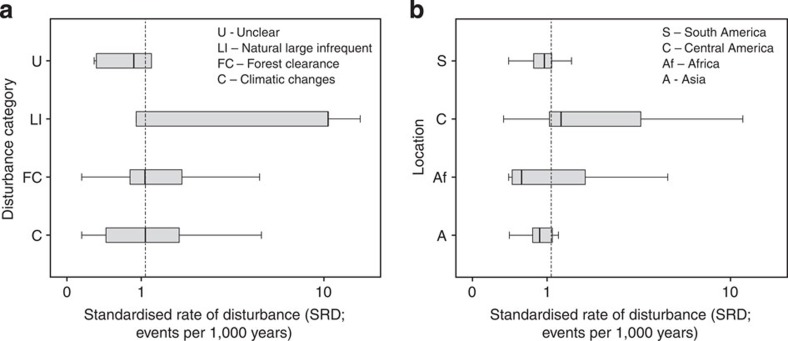
Composite figure showing the standardized rate of disturbance (SRD) of different disturbance categories and locations. Box plots displaying the relationship between the SRD and (**a**) different grouped disturbance categories and (**b**) geographical location groups. (See Fig. 2 legend for details on notation and sample sizes). The SRD is plotted on a logarithmic scale to accommodate the variability in the data set. The vertical line in **a** and **b** represents the median SRD for the entire data set, that is, 1.072 disturbance events per 1,000 years. Shaded areas on **a** and **b** represent the interquartile range and the whisker lines the 95% confidence intervals for each category.

The standardized rate of disturbance events, SRD, which was calculated by dividing the number of disturbances in a site by the approximate time over which they occurred, giving a rate per 1,000 years (Table 1) allows investigation of whether frequency of past disturbance contributes to the pattern of RR observed across these tropical forests. Of all covariates tested, the SRD displayed the most significant relationship with RR (linear mixed-effects model, *P* value<0.001, *n*=283; Table 3) (Fig. 2c), indicating that forests that had experienced a higher frequency of disturbance events in the past tended to recover more rapidly after each subsequent perturbation. Further analysis demonstrated that of the four different categories of disturbance recorded, large infrequent events occurred at the greatest rate (median=10.444 events per 1,000 years, *n*=13) and forest clearance at the lowest (median=1.072 events per 1,000 years, *n*=87) (Fig. 4a; Table 4). In terms of geographical location, forested sites in the African tropics experienced the least frequent disturbance in the past (median=0.578 events per 1,000 years, *n*=29) and Central American forests the most (median=1.278 events per 1,000 years, *n*=111) (Fig. 4b; Table 4). Although these patterns have emerged, none of the locations or disturbance categories proved significantly different to each other in their SRD (except for climatic disturbance and forest clearance; Table 4), suggesting, for example, that forests in Central America have not experienced considerably more disturbance events per millennium than the other tropical regions in the past. Greater and more consistent sample sizes across groups would help to clarify these differences.

## Discussion

Results from this meta-analysis show that tropical forests have been disturbed by a variety of factors over the Late Glacial and Holocene periods, and recovery has proceeded at varying rates. Our analyses indicate that for most tropical forest ecosystems, the length of time taken to re-establish forest to values *c.* 95% of former arboreal abundance takes >200 years. This finding is supported by palaeoecological studies independent of this analysis (lacking inclusion requirements), for example (ref. 8). Only after approximately 20% of disturbance events did forest recover within the 42 years reported as the average for complete recovery in tropical and boreal forests across the world^9^. Length of time is an important distinction to make because while there are a number of studies that suggest that any form of disturbed (often referred to as secondary) forest has less ecological value than primary forests^10^,^11^, few of these have examined forests that have been recovering for >200 years and therefore are unlikely to be comparing like-with-like but rather a forest ecosystem in the early stages of succession. This relatively extended period required for recovery is relevant to contemporary forestry management where periods allocated for forest re-growth post-logging are rarely ecologically suitable^12^ and practices are underpinned by inadequate and biased knowledge^13^. Incorporating a longer rotation period into forest management plans, to allow for enhanced regeneration, may improve forest productivity.

Significant differences in recovery rates suggest that certain factors will influence the speed of tropical forest restoration. The result that recovery is fastest following natural disturbances, such as hurricanes, is somewhat intuitive; these are processes that have been occurring throughout the earth’s history and considered to play an important role in ecosystem dynamics^14^. Despite being labelled as large infrequent disturbances, an ecological term that illustrates the greater intensity, duration and spatial extent of these perturbations compared with those that ‘typically’ affect an ecosystem^15^, it is also the type of perturbation that has occurred with the greatest frequency (SRD) in the past (Fig. 4a). This further supports one of the main conclusions of this study that these ecosystems recover most rapidly after exposure to natural disturbances, occurring at frequencies through time of ecological relevance, that is, events per thousands of years (see for example, the tens of years and novel forms of disturbance pervading many of these ecosystems today), which results in their greater ability to respond dynamically, manifest here in an increasing rate of recovery, and inferred resilience. Similar findings arose from a study investigating recovery rates in overexploited marine populations^16^; moderate levels of stock depletion over an extended period, that is, >50 years, led to the adaptation of populations to the historical fishing regime, enhancing recovery rates and thus promoting resilience. This result lends further support, from a disparate biome to the fundamental concept of ecological adaptation, whether through life-history changes^16^ or ecological sorting/habitat filtering^17^, over long-term time scales. For these tropical forest ecosystems, this result implies that designing anthropogenic forest disturbances that more closely mimic natural disturbance regimes will result in a lesser decline in the abundance of trees in the landscape and may markedly reduce the time required and resources needed for forest regeneration. The slow recovery rates recorded following human-induced burning are of particular concern, given that this is the favoured mode of conversion to agriculture in tropical forests; it is also sometimes adopted by conservation managers to enhance diversity through invoking a particular disturbance regime^18^.

The slower recovery rate apparent in Asia and South America compared with Central America and Africa may be attributable to current abiotic drivers, such as soil fertility^4^ and climate^19^, and biotic influences such as vegetation life histories determining functional group composition^5^, tree species diversity^20^ and age structure^7^; variables inherently linked to the regime of past disturbance events^4^,^21^. The species and functional diversity of these tropical forest communities, the initial state of the forest pre-disturbance and the relative severity of the disturbance events^3^, for example the resultant impact on seed availability and seedling vulnerability^22^, were not explicitly considered in this study (in the most part due to data limitations), though all may play a significant role in shaping the rates and patterns of recovery observed here^23^,^24^. Future work, investigating in more detail the impact of these abiotic and biotic components (via the development of a universal independent proxy in the case of disturbance magnitude), would extend understanding of the causal reasons underlying this study’s findings and provide further knowledge on the ecology of tropical forests, as well as information for application in ecosystem management and restoration. Nonetheless, despite the relatively coarse temporal and spatial scale of the analyses performed here, findings provide many valuable insights for the sustainable use of tropical forest resources, not least estimates of the time required for the reforestation of these pivotal ecosystems. For the development of successful ecosystem service markets, trading in, for example, carbon credits resulting from effective forest restoration, this information is sorely lacking and urgently required^25^.

In addition, this study has been able to test for the first time the hypothesis that increased past exposure to perturbation (in particular frequency of events per 1,000 years, Fig. 2c) leads to the evolution of adaptations that enable forested ecosystems across the tropics to respond more dynamically to future disturbance. As well as the abiotic and biotic factors mentioned previously, the faster rates of forest re-growth in Africa and Central America may result from more extensive histories of disturbance in those regions. Past perturbations relevant to the ecological context of this meta-analysis may include human disturbance resulting from large-scale forest-based ancient civilizations^26^,^27^,^28^ and extreme weather events, for example, droughts in Central Africa^29^ and hurricanes in Central America^30^.

This meta-analysis has demonstrated disturbance-recovery dynamics over long-term time scales, revealing increasing recovery rates with higher frequency of disturbance (Fig. 2c). Such a result runs counter to recent studies modelling ecosystem resilience to contemporary forms and intensities of disturbance, namely human induced over short time scales, which, to a great extent, are far from mimicking natural disturbance regimes^13^,^31^. For example, refs 32, 33 identify slowing recovery rates with increasing disturbance as a robust indicator of a loss of resilience in different systems. It is important that a distinction is drawn between the types of disturbance experienced in the past versus today, with the impacts of contemporary regimes, and forest responses, yet to be fully observed. Accounting for both hypotheses on resilience may require careful consideration of both the type of perturbation and the temporal and spatial context of the ecosystem, which determines its ecological memory and consequently the disturbance and recovery dynamics. For understanding resilience, this study has demonstrated how crucial it is to adopt a long-term view in analysis.

## Methods

### Chronological calibration

Various procedures were used to date the published diagrams. Where age-depth models were not present in the published study, the dating programme, *clam*^34^, in R35 (ref. 35), was used to construct the best-fitting age-depth model for the reported ages. Similarly, *clam* was used to calibrate dates reported in ^14^C radiocarbon years. Where data were not available in the reference for surface dates, a default of −55 Cal. yr BP, with error 1, was used, in line with conventional dating of modern samples^36^. In the absence of age uncertainties for a radiocarbon date, an average of the value for dates on either side was used, or in the case of basal samples, the youngest date reported. If no age uncertainties were reported, a default of 50 years was used, for example in ref. 37. IntCal09 and SHCal04 curves were used for Northern and Southern Hemisphere age-depth model construction and calibrations, respectively, except where the latter had dates older than 10,522 ^14^C radiocarbon years, in which case the IntCal09 curve was employed. Calibrated ages are reported as the average of the 95.4% two sigma (2*σ*) calibrated age range that obtains the greatest relative area under the probability distribution curve.

### Measurements and notation

Table 1 documents the focal features and variables extracted from pollen diagrams, used in calculations of recovery rates. Details of the disturbance types, and associated indicators, recorded from published palaeoecological studies are displayed in Table 2.

### Data acquisition and analysis

The response metric, RR, calculated as percentage forest pollen increase per unit time from the fossil pollen diagrams selected for this meta-analysis, provides a basic measure of forest recovery. RR is measured as an average across the entire period of forest pollen increase (representing forest re-establishment), until the maximum increase in percentage of forest pollen post-decline is achieved, before a stabilizing point or further decline. It therefore incorporates any non-linear components of the trajectory, which may result, for example, from low-level perturbations during the period of recovery; it is, however, the overall rate at which the forest is responding to each disturbance event that is the variable of interest here. Datathief III^38^ was used to extract RR components from the published fossil pollen diagrams, to reduce potential bias resulting from visual interpretation and assist in the calculation of an average rate over the entire period of forest re-establishment.

The species or functional diversity of the individuals contributing to this summed forest value was not recorded, in part due to lack of reporting of such information across selected studies. Thus, the computed RR does not take into account the biotic character, for example, primary versus secondary taxa, and turnover in the recovering vegetation, both of which form important components of ecological resilience and may influence the speed at which the ecosystem recovers from perturbation^24^. The focus of this meta-analysis is rather on the RR of forest, as defined uniquely in each individual study (see Supplementary Data 1 for details of studies), representing the speed and extent to which this ecological unit re-grows relative to its pre-disturbance abundance, with the assumption that vegetation re-establishment, as inferred through the proxy of fossil pollen, is indicative of sustained ecosystem functionality. Under this criteria, a variety of forest types, ranging from full canopy cover to some fraction of that (indicated by F_pre_ values of individual events; see Supplementary Data 1), are included in this meta-analysis.

Where emergent variables measured from pollen diagrams demonstrated a skewed pattern, such as F_max_, and sample sizes were quite different between groups within variables, for example, disturbance type, non-parametric descriptive statistics were used to illustrate trends in the data.

The distributions of the response variable (RR) and predictor covariates, for example SRD, were observed and transformations performed, resulting in logarithms of RR and SRD being used in the final analyses. Multiple regression analyses were run in R^35^, using the *nlme* package^39^, to investigate the relationship between RR and the range of recorded variables. Model I (equation 1) represents the linear mixed-effects model used in the analysis of RR:


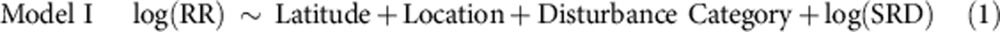


(See Table 3 for model output.) Site ID was included as a random effect, providing a unique identifier for each fossil pollen record, to obviate potential bias resulting from pseudoreplication and to enable exploration of overall patterns in the data set without weighting each effect size by their associated group size (that is, number of events per pollen record); this was considered an inappropriate transformation in this meta-analysis given that effect sizes within each group varied greatly. Several covariates were excluded from the final multiple regression model (Model 1, Eq. 1), after they demonstrated an insignificant relationship with the response variable, log(RR) (linear mixed-effects model, *P* value>0.1, *n*=283), when considering their partial slopes: altitude (linear mixed-effects model, *P*-value=0.625, *n*=283) and sequential number of the disturbance event (linear mixed-effects model, *P*-value=0.728, *n*=283), both square-root transformed. Through manipulation of Model I (Eq. 1), comparisons were made of RR across location and disturbance category groups. To investigate the emergent significant relationship between RR and SRD (linear mixed-effects model, *P*-value<0.0001, *n*=283; Table 3), the degree of correlation between SRD and the location and disturbance category groups was tested using the following model (Eq. 2):


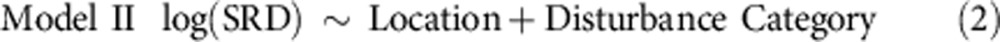


(See Table 4 for model output.) Site ID was included as a random effect (proving a more important component of this model due to the nature of SRD measurements). A third model, including log(T_rec_) as a covariate (Model III), demonstrated a significant relationship with log(SRD) (linear mixed-effects model, *P*-value<0.01, *n*=283); however, since T_rec_ is used in the calculation of RR, this finding is not independent of the findings from Model I. In addition, disturbance events are assumed to occur randomly through time in each location, not as a function of the time spent in or level of recovery of the forest ecosystem.

## Author contributions

L.E.S.C., S.A.B. and K.J.W. designed the study; L.E.S.C. collected and analysed the data; L.E.S.C., S.A.B. and K.J.W. wrote the manuscript.

## Additional information

**How to cite this article:** Cole, L. E. S. *et al.* Recovery and resilience of tropical forests after disturbance. *Nat. Commun.* 5:3906 doi: 10.1038/ncomms4906 (2014).

## Supplementary Material

Supplementary Data 1 All published studies from which data was extracted and variables recorded from diagrams.

## Figures and Tables

**Table 1:** Main features and variables extracted from published pollen diagrams for recovery rate calculations.

**Variable**	**Description**	**Notation**
*Independent variables*		
Disturbance type	Factor causing impact on forest vegetation, shown on pollen diagram or referred to in the text	See Table 2 for categories
Geographical attributes	Potential influencers of forest ecology/disturbance response	Location, altitude, latitude, longitude
Standardized rate of disturbance events (SRD)	Average number of disturbance events in a site per 1,000 years	SRD=(*n*/(T_1,pre_–T_*n*,max_))*1,000
*Response variable & measurements*		
Recovery Rate (RR)	Rate of increase in forest abundance relative to degree of disturbance-induced change, that is, % increase in forest pollen abundance per year in relation to pre-disturbance level	RR=(((F_max_–F_min_)/(F_pre_–F_min_))*100)/T_rec_
Forest abundance maximum pre-disturbance (%)	Percentage of forest pollen at maximum point pre-decline (that is, baseline forest pollen percentage)	F_pre_
Forest abundance minimum at disturbance (%)	Percentage of forest pollen at minimum point during disturbance event	F_min_
Forest abundance at maximum recovery (%)	Percentage of forest pollen at point of maximum recovery (before a stabilizing point or further decline)	F_max_
Time period of recovery (years)	Time period from maximum reduction to maximum recovery	T_rec_
Forest abundance decline (%)	Percentage decline in forest pollen from F_pre_	FD=((F_pre_–F_min_)*100)/F_pre_

RR=recovery rate; SRD=standardized rate of disturbance.

Forest recovery is described as the maximum increase in percentage of forest pollen after a decline, before a stabilizing point or further decline. (*Forest abundance* is used as a crude descriptor of past vegetation extent reconstructed from fossil pollen, but is not representative of a quantifiable forest area.) (*n*=number of disturbance events).

**Table 2:** Disturbance types and indicators (proxies) extracted from published palaeoecological studies.

**Disturbance source**	**Disturbance type**	**Proxy**
Natural	Climate (C)Precipitation (CP)Sea-level rise (CS)	Oxygen isotopes, fire (low levels, not linked to human presence), magnetic susceptibility, lithologyRainfall, monsoon strength variation, climate drying (CD)Sea level
	Large infrequent (LI)	Hurricane (LI-H), landslide (LI-L), fire (LI-F), volcano (volcanic ash) (LI-V)
Human (FC)	Burning (B)	Microfossil & macrofossil charcoal
	Forest clearing (FC)	Temporary, predominantly resulting from shifting cultivation (SC), or more permanent, generally selective clearing, or not described (FC) signified by for example, fruit trees, *Poaceae*, & disturbance indicators/secondary forest taxa, for example, *Arenga* and *Macaranga*, or magnetic susceptibility
	Agriculture (Ag)	Agricultural indicators, for example, fruit trees—*Ficus*, crops—*Poaceae*
Unclear	(U)	Disturbance indicators present but specific type not clarified

Disturbance types and proxies (used to identify the former) were extracted either from published pollen diagrams or from the associated text. Abbreviations for the different disturbance types are given in parentheses. Smaller natural perturbations such as tree falls, though not explicitly defined due to the difficulty of identification through fossil pollen analysis, may also contribute to large infrequent (LI) disturbance events. (See Supplementary Data 1 for the full record of disturbance types referred to here.)

**Table 3:** Results of multiple regression analysis using Model I.

**Variables**	**Coefficient**	**s.e.**	***P*****-value**
Intercept	−1.597	0.252	0.000
Latitude	−0.020	0.012	0.111
Location—Africa	0.856	0.315	0.009*
Location—Central America	0.794	0.351	0.027*
Location—South America	0.252	0.223	0.264
Disturbance category—FC	0.296	0.187	0.115
Disturbance category—LI	0.764	0.423	0.072
Disturbance category—U	0.936	0.346	0.007*
Log(SRD)	0.914	0.082	0.000†

Output for Model I: log(RR)~Latitude+Location+Disturbance Category+log(SRD), *n*=283. Location—*Asia* and Disturbance Category—*Climate* were set as the reference groups for the initial model output.

^*^Subgroups within categorical variables that are significantly different from the reference set.

^†^SRD is a highly statistically significant independent predictor of RR.

**Table 4:** Results of multiple regression analysis using Model II.

**Variables**	**Coefficient**	**s.e.**	***P*****-value**
Intercept	0.213	0.200	0.291
Location—Asia	−0.308	0.307	0.320
Location—Central America	0.296	0.283	0.298
Location—Africa	0.229	0.414	0.519
Disturbance category—LI	0.021	0.084	0.804
Disturbance category—C	−0.103	0.032	0.002*
Disturbance category—U	−0.075	0.046	0.106

Output for Model II: log(SRD)~Location+Disturbance Category, *n*=283. Location—*Africa* and Disturbance Category—*Forest Clearance* were set as the reference groups for the initial model output.

^*^The SRD caused by climatic impacts (C) is significantly different from that caused by forest clearance (FC).
